# Google Trends Analysis of Interest in Pediatric Myopia-Control Spectacle Lenses Following FDA Authorization of Essilor Stellest

**DOI:** 10.7759/cureus.110127

**Published:** 2026-06-02

**Authors:** Arbaaz S Gill, Connor C Tseng, Patrick S Burke, Laura S Kueny

**Affiliations:** 1 Ophthalmology, George Washington University School of Medicine and Health Sciences, Washington, DC, USA; 2 Ophthalmology, Children's National Hospital, Washington, DC, USA

**Keywords:** defocus incorporated multiple segments, digital epidemiology, essilor stellest, fda authorization, google search trends, interrupted time series, myopia control, myopia-control spectacle lenses, pediatric myopia, public interest

## Abstract

Purpose: To quantify changes in US public interest in pediatric myopia-control spectacle lenses surrounding the U.S. Food and Drug Administration (FDA) authorization of Essilor Stellest (Essilor, Charenton-le-Pont, France) (September 25, 2025).

Methods: Weekly U.S. Google Trends relative search volume (RSV) data were collected for 2025 for parent-facing and brand-specific pediatric myopia-control spectacle lens search terms, with MiSight-related searches analyzed as a contemporaneous comparator. Search terms were selected a priori to capture both direct interest in the Stellest product and broader public interest in pediatric myopia-control spectacle lenses. A composite interest index was constructed by averaging RSV values for the following terms: “stellest”, “stellest lenses”, “essilor”, “essilor stellest”, “myopia control glasses”, “pediatric myopia glasses”, and “HALT lens”. To account for general seasonality in children’s eyewear searches, a baseline index was generated using the mean RSV of “kids glasses”, “children’s glasses”, and “eyeglasses for kids”, and a normalized interest ratio (composite ÷ baseline) was calculated. Pre- and post-authorization differences were compared using two-sided p-values.

Results: From 2025-01-05 to 2025-10-26 (37 pre- and six post-weeks), mean composite interest rose from 9.39 pre to 18.6 post (absolute +9.21, +98%; p = 0.024). The mean ratio to baseline increased from 0.353 to 0.694 (absolute +0.34, +96.3%; p = 0.016). MiSight-related searches remained stable over the same period, supporting a modality-specific response to Stellest authorization.

Conclusions: Public search interest in pediatric myopia-control spectacle lenses increased markedly following FDA authorization of Essilor Stellest, with normalization suggesting growth beyond seasonal effects. These findings demonstrate increased online search activity surrounding pediatric myopia-control spectacles after authorization, which may help clinicians anticipate heightened patient and parent inquiries, although search interest does not directly reflect awareness, clinical adoption, or prescribing behavior.

## Introduction

Background

Pediatric myopia prevalence is increasing globally, with early-onset disease associated with a higher lifetime risk of myopia and vision-threatening ocular complications [1]. In response, interest in myopia-control interventions has grown, including pharmacologic therapies (low-dose atropine), contact lens-based approaches (orthokeratology and MiSight (CooperVision, San Ramon, CA)), and spectacle lens technologies [2]. MiSight, a daily soft contact lens approved by the U.S. Food and Drug Administration (FDA) in 2019, is an established modality used in pediatric myopia control [3]. However, its use may be limited in younger children due to challenges with lens handling, adherence, and caregiver concerns regarding hygiene and safety [4].

Regulatory and technological context

The Essilor Stellest lens (Essilor, Charenton-le-Pont, France) is a spectacle-based optical design that combines distance correction with peripheral defocus-inducing aspherical lenslets, which are proposed to create a distributed myopic defocus signal intended to slow axial elongation while maintaining clear central vision [5,6]. In comparison, MiSight 1-day contact lenses deliver myopic defocus through concentric treatment zones within a soft contact lens platform [3]. On September 25, 2025, the U.S. FDA authorized marketing of Essilor Stellest lenses, the first spectacle lenses approved in the United States for slowing pediatric myopia progression [5,6]. In 2025, the FDA did not approve SYD-101, a low-dose atropine formulation evaluated in the CHAMP trial, citing insufficient evidence of efficacy for slowing myopia progression [7,8]. This regulatory outcome provides context for the emergence of Stellest as a newly authorized spectacle-based option within the U.S. myopia-control landscape.

Rationale for the study

As parents are the primary decision-makers for children’s eye care and purchasers of pediatric eyeglasses [9], measuring search activity provides a useful proxy for parental information-seeking behavior and public interest in emerging technologies [10]. Google Trends offers anonymized, relative search interest over time and has become a powerful tool for tracking public awareness of medical technologies [11]. Understanding how regulatory authorization influences public information-seeking behavior is increasingly important as myopia-control options diversify and shared decision-making becomes central to pediatric eye care.

## Materials and methods

Study design and data source

We conducted an observational pre-post time-series analysis using weekly U.S. Google Trends relative search volume (RSV) data [11]. As this study used publicly available, anonymized, and aggregated data, it was exempt from institutional review board approval. The study was conducted and reported in accordance with the STROBE (Strengthening the Reporting of Observational Studies in Epidemiology) guidelines for observational studies [12].

Search term selection and exposure definition

Search terms were selected a priori to capture parent-facing interest in spectacle-based pediatric myopia-control lenses, including both direct Stellest-related searches and broader descriptive queries: "Stellest", "Stellest lenses”, “Essilor Stellest", “myopia control glasses”, “pediatric myopia glasses", and “HALT lens”. A preliminary review in Google Trends was used to confirm sufficient search volume for inclusion. Averaging across multiple related search terms was intended to reduce volatility associated with individual query fluctuations and better capture aggregate public interest in spectacle-based pediatric myopia control. The general brand term “Essilor” was included to capture brand-associated search activity but was also evaluated in sensitivity analyses due to its broader scope. Terms related to contact lens-based interventions (e.g., orthokeratology), pharmacologic therapies (e.g., atropine), refractive surgery, and adult myopia were excluded to isolate interest in spectacle-based pediatric myopia-control options.

Outcome measures

The composite interest index was defined as the weekly mean RSV across included Stellest-related terms. To account for seasonal variation in general pediatric eyewear demand, a composite baseline was defined as the mean RSV of “kids glasses”, “children’s glasses”, and “eyeglasses for kids”. For the primary Stellest analysis, the ratio to baseline was computed as composite ÷ baseline for each week.

Comparator and sensitivity analyses

To contextualize whether observed changes in search interest were specific to the FDA authorization of Stellest rather than reflecting broader trends in myopia control interest, we conducted a secondary analysis of MiSight-related search terms over the same study period (January 5 to October 26, 2025). MiSight composite interest was defined as the weekly mean RSV for “MiSight”, “MiSight contact lens”, “MiSight myopia”, and “MiSight lenses”. As Google Trends normalizes RSV within each query, the Stellest and MiSight series were analyzed independently, and comparisons were restricted to within-modality temporal changes rather than direct between-modality comparisons. As a sensitivity analysis, we examined the temporal trajectory of the standalone term “Essilor” to assess whether observed patterns in the Stellest composite could be driven by broader brand-level search activity.

Time periods and statistical analysis

The post-intervention period was defined to begin the week of September 21, 2025, which includes the September 25 FDA authorization. Thus, six weeks (September 21 to October 26) were considered post-authorization. For each modality, the mean weekly RSV in the pre-authorization period was compared with the post-authorization period. Differences in weekly means were assessed using two-sample t-tests, with two-sided p-values reported (α = 0.05) [13,14]. All analyses were conducted in R (version 4.4.3; R Foundation for Statistical Computing, Vienna, Austria), with data extraction and analysis performed in November 2025.

## Results

Overall trends in public search interest

**Figure 1:**
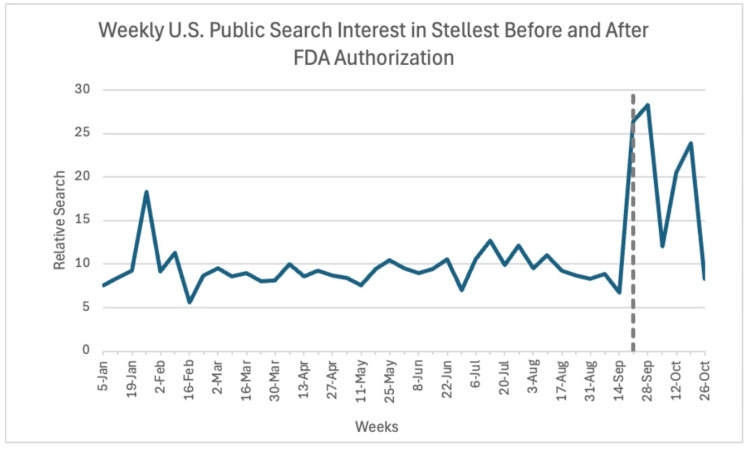
Weekly U.S. Google Trends relative search volume (RSV) for Stellest-related pediatric myopia-control spectacle lens terms from January 5 to October 26, 2025. The dashed vertical line indicates the week of U.S. Food and Drug Administration authorization (September 21, 2025). Weekly RSV values are shown over time.

**Figure 2:**
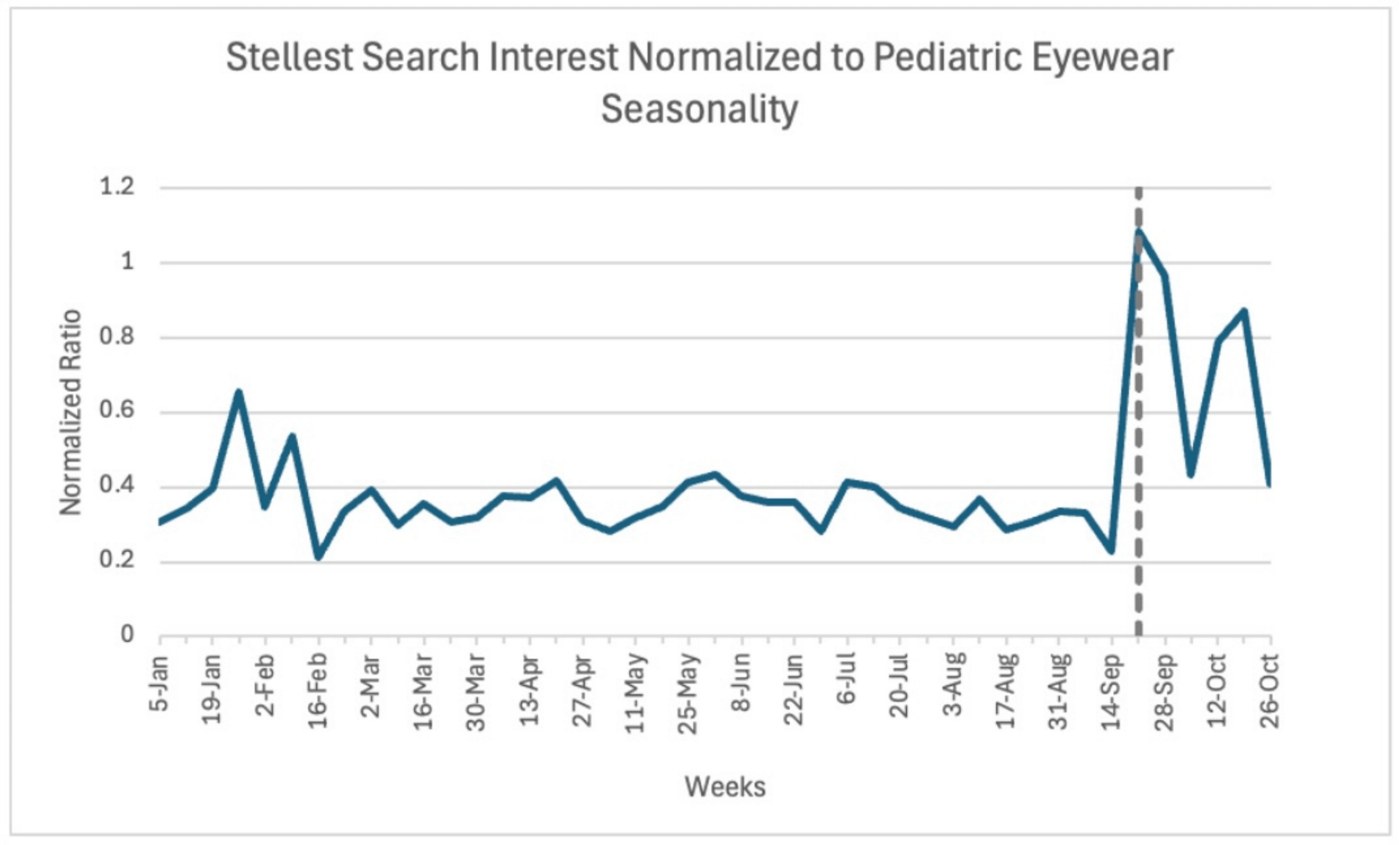
Ratio of Stellest composite search interest to a baseline of general pediatric eyewear searches ("kids' glasses”, “children’s glasses”, and “eyeglasses for kids”) from January 5 to October 26, 2025. The ratio was calculated to adjust for seasonal variation in children’s eyewear demand. The dashed vertical line indicates the week of U.S. Food and Drug Administration authorization (September 21, 2025).

Across 37 pre-authorization and six post-authorization weeks (January 5 to October 26, 2025), the mean weekly search interest increased following FDA authorization of Essilor Stellest lenses [5,6]. An increase in weekly relative search volume was observed beginning the week of September 21, 2025, which includes the FDA authorization period. Search interest remained higher in the post-authorization period through late October (Figures 1, 2). Statistically significant differences were observed for both the composite interest and ratio-to-baseline measures (p < 0.05 for both comparisons).

Primary outcome: composite interest and ratio-to-baseline

The composite search interest (mean of Stellest-related terms) increased from 9.39 pre-authorization to 18.6 post-authorization, representing an absolute increase of +9.21 and a 98% relative increase (p = 0.024) (Figure 1).

The ratio-to-baseline measure also rose from 0.353 to 0.694, an absolute change of +0.34 and a 96.3% relative increase (p = 0.016) (Figure 2).

Sensitivity and comparator analyses

Examination of the standalone term “Essilor” did not exhibit a discrete inflection or sustained increase during the FDA authorization period.

**Figure 3:**
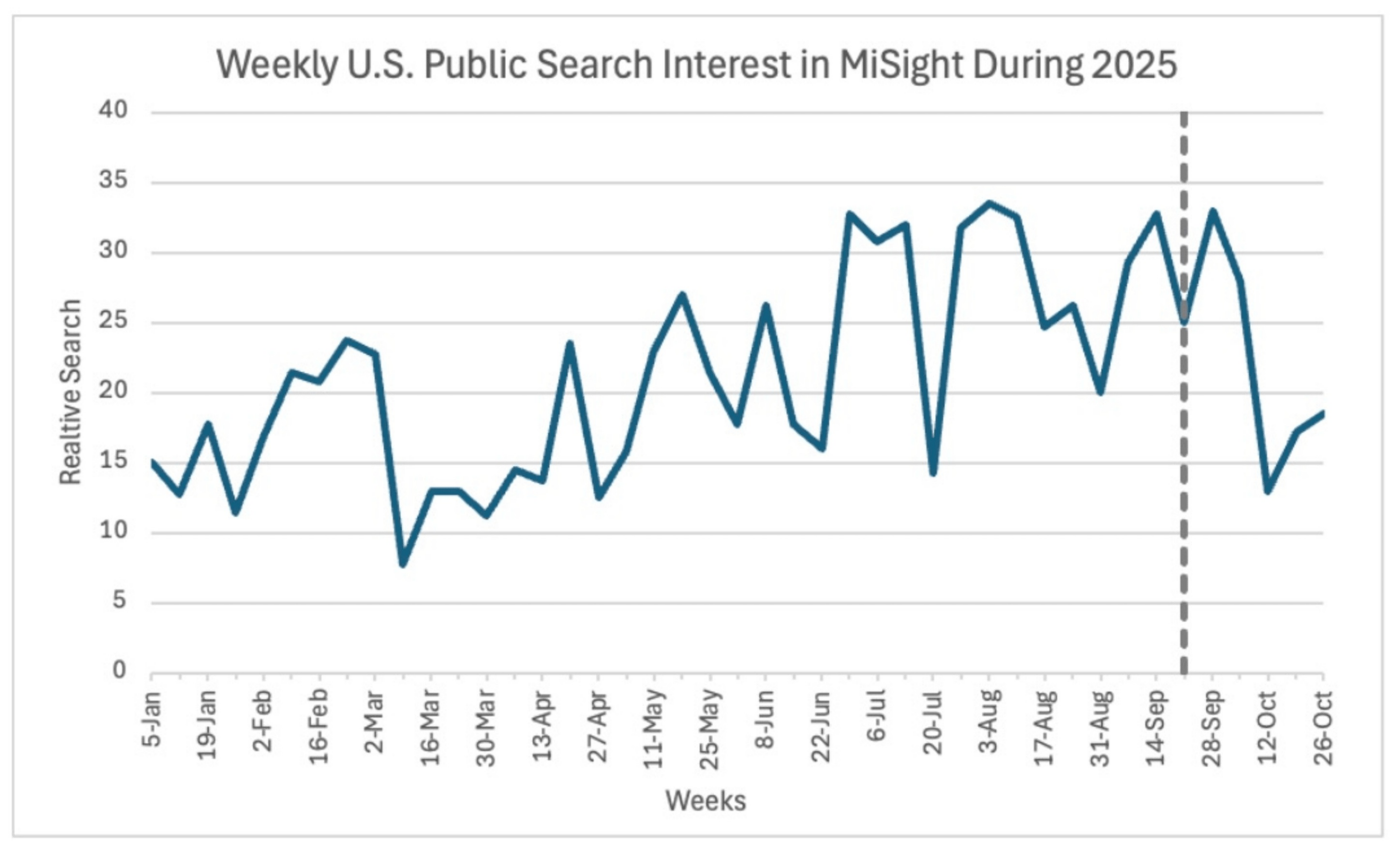
Weekly U.S. Google Trends relative search volume (RSV) for MiSight-related pediatric myopia-control contact lens terms from January 5 to October 26, 2025. The dashed vertical line indicates the week of U.S. Food and Drug Administration authorization for Stellest (September 21, 2025). RSV values are shown over time.

Over the same study period, MiSight-related search interest did not demonstrate a significant change between the pre- and post-authorization periods (p = 0.6733), with weekly relative search volume remaining stable throughout 2025 (Figure 3).

## Discussion

This analysis demonstrates a substantial rise in U.S. public search interest in pediatric myopia-control spectacle lenses following the FDA authorization of Essilor Stellest. The temporal alignment between the authorization date of September 25, 2025, and the observed inflection point suggests a potential association between regulatory approval and heightened online search activity (Figure 1). Such regulatory milestones are often accompanied by heightened information-seeking behavior, particularly when a technology represents a first-in-class option for a common pediatric condition. Importantly, the statistically significant increases in both absolute composite interest and the ratio-to-baseline indicate that this post-authorization surge was not driven solely by seasonal eyewear demand but may reflect increased online search interest temporally associated with FDA authorization.

Search interest in myopia-related topics spans multiple related terms with varying popularity, reflecting heterogeneity in how the public seeks information about myopia control [15]. Aggregating these terms reduces volatility associated with individual queries and provides a more stable proxy for overall interest. Figure 2 presents the same composite interest normalized to a baseline of general pediatric eyewear searches, accounting for predictable seasonal fluctuations in children’s glasses demand. The similar trends between Stellest in Figure 1 and Figure 2 indicate that the post-authorization increase was not driven solely by routine eyewear purchasing cycles or back-to-school seasonality. Rather, the parallel trends suggest increased technology-specific search interest temporally associated with FDA authorization. Normalizing this composite against a pediatric eyewear baseline provides an additional safeguard against misattributing seasonal effects to technology-driven interest, and the resulting ratio-to-baseline metric serves as a conservative measure of relative growth in public interest rather than absolute demand [11,16]. Together, these findings demonstrate a substantial increase in US online search interest in pediatric myopia-control spectacles following FDA authorization.

Several contextual factors likely contributed to the magnitude of the observed increase. In the United States, EssilorLuxottica supported the Stellest launch through coordinated marketing and educational efforts directed at pediatric eye-care practices and parent-facing platforms. Industry webinars and clinician training modules were disseminated shortly after authorization, and coverage by optometry- and pediatrics-focused media outlets amplified early public awareness [17-19]. Concurrently, Essilor’s social media messaging emphasized Stellest as the first-and-only FDA-authorized spectacle lens for slowing pediatric myopia progression, likely reinforcing public interest [20].

Comparing Stellest with existing myopia-control options provides additional context for interpreting parental behavior. MiSight 1-day contact lenses, FDA-approved in 2019, remain an effective and widely adopted intervention but deliver myopia-control signals through discrete treatment zones within a contact lens, requiring daily lens insertion and removal and carrying a small but real risk of contact lens-related ocular infections, which may deter some families [3]. In contrast, Stellest delivers a myopia-control optical signal through a spectacle-based lenslet design embedded within a conventional eyeglass platform, offering a lower-burden alternative that aligns with existing eyewear habits, particularly for younger children or families hesitant about contact lenses [5,6].

Over the same time period, MiSight did not demonstrate a significant change in search interest between the pre- and post-authorization periods (p = 0.6733), in contrast to the marked post-authorization increase observed for Stellest (Figure 3). Given MiSight’s earlier FDA approval in 2019 and its established presence in clinical practice, this relative stability in search interest was expected and serves as a contextual comparator, supporting the interpretation that the observed surge in Stellest searches may represent a modality-specific pattern temporally associated with recent FDA authorization rather than a broader increase in myopia-control-related search activity.

Regulatory developments in 2025 may have further sharpened public focus on spectacle-based myopia control. In the same year that Stellest received FDA authorization, the FDA declined approval of SYD-101, a low-dose atropine formulation investigated for pediatric myopia control [7,8]. The absence of an FDA-approved pharmacologic option may have intensified attention toward Stellest as the only newly authorized modality in the US market.

Because parents are the primary decision-makers for pediatric eye care, interpreting Google Trends data through the lens of parental behavior is particularly relevant. Parents express variable awareness and concern about myopia progression and control strategies, with substantial gaps in knowledge about available treatment options and varying perceptions of risk and burden associated with different modalities [21]. Against this backdrop, the ratio-to-baseline findings shown in Figure 2 are clinically meaningful, as they suggest that increased searches reflect targeted interest in myopia control rather than routine eyewear replacement.

Limitations

This study has limitations. Google Trends provides relative rather than absolute search volumes and does not directly measure clinical adoption or prescribing behavior directly. Search activity may be influenced by concurrent marketing efforts or media coverage. Google Trends data may also vary slightly depending on the date of extraction and query normalization procedures. Additionally, the use of simple pre/post comparisons does not account for potential temporal dependence between weekly observations. Because FDA authorization occurred late in 2025, the post-authorization window was limited to six weeks. Accordingly, these findings should be interpreted as an early observational assessment of post-authorization search behavior rather than a long-term trend analysis. Future studies incorporating longer post-authorization periods and formal segmented regression analyses may further clarify the durability and trajectory of public interest. We attempted to include SYD-101 as a pharmacologic comparator; however, Google Trends returned insufficient search volume to generate a weekly time series. Future work integrating prescription data, insurance claims, or clinic-level adoption metrics could help determine whether increased online search interest translates into sustained clinical uptake.

## Conclusions

Public search interest in pediatric myopia-control spectacle lenses rose markedly following FDA authorization of Essilor Stellest. These findings suggest increased online information-seeking behavior surrounding pediatric myopia-control spectacles after authorization. Clinicians may anticipate greater patient and parent interest in spectacle-based myopia-control options and should be prepared to discuss available interventions, including MiSight contact lenses, orthokeratology, and low-dose atropine, within an evidence-based counseling framework.
